# Case Report: Varyingand Unique Symptomatic Presentations of Congenital Intrahepatic Portosystemic Venous Shunts

**DOI:** 10.3390/reports9030236

**Published:** 2026-07-22

**Authors:** Christopher Stevens, Eric Wallace, Chaitanya Ahuja

**Affiliations:** 1 Department of Surgery, Baylor College of Medicine, Houston, TX 77030, USA; 2 Department of Radiology, Louisiana State University Health Sciences Center-Shreveport, 1501 Kings Highway, Shreveport, LA 71103, USA; eric.wallace@lsuhs.edu (E.W.);

**Keywords:** intrahepatic portosystemic venous shunts (IPSVSs), interventional radiology, embolization

## Abstract

**Background and Clinical Significance**: Intrahepatic portosystemic venous shunts (IPSVSs) are rare hepatic vascular malformations that occur when there is an abnormal communication between the hepatic and portal veins. IPSVSs can be acquired or congenital, with the latter being the most common. **Case Presentation**: In this manuscript, we report two cases of symptomatic IPSVSs that were likely congenital in etiology and varied in presentation. The shunts were diagnosed using ultrasound, CT, and MRI, followed by successful treatments with transcatheter embolization procedures. **Conclusions**: This report highlights the high degree of symptomatic variance that can be seen in patients with symptomatic IPSVSs, as each case presented with different symptomatic features, while also reinforcing the notion that the use of ultrasound, CT, and MRI is of high importance when trying to diagnose IPSVSs. In addition, this article also adds to the existing literature that transcatheter embolization is a valuable therapeutic approach for symptomatic IPSVSs.

## 1. Introduction and Clinical Significance

Intrahepatic portosystemic venous shunts (IPSVSs) are rare hepatic vascular malformations that occur when there is an abnormal communication between the hepatic and portal vein [1]. IPSVSs can be acquired or congenital, with the latter being the most common [1]. Acquired IPSVSs are normally less than 2 mm in size and are often a result of cirrhosis or trauma [2,3,4]. Contrarily, congenital IPSVSs tend to be larger in nature and not associated with cirrhosis [5]. Congenital IPSVSs may present incidentally or with complications ranging from metabolic abnormalities to pulmonary vascular disease and hepatic lesions [6]. According to Simona Juncu et al., spontaneous portosystemic shunts are increasingly recognized as markers of advanced portal hypertension and are associated with several clinically important complications, the most important being hepatic encephalopathy. Other associations include variceal bleeding, portal vein thrombosis, and portopulmonary hypertension [7]. As a result, awareness of the diverse manifestations of these shunts is important for timely diagnosis and appropriate management.

Neonates with congenital IPSVSs can sometimes present with hyperammonemia, cholestasis, and/or hypergalactosemia [1]. Incomplete involution of the vitelline venous network is likely the main reason for the development of congenital IPSVSs [8]. By the fifth week in utero, the embryonic venous system comprises three major pairs of veins—the vitelline (known also as the omphalomesenteric veins), umbilical veins, and cardinal veins. Initially, the two vitelline veins drain into the sinus venosus, and by the end of the 4th week they form a vitelline venous network around the duodenum [8]. The vitelline veins are connected through a plexus of veins that give rise to hepatic sinusoids that involute during the 8th week to form the intrahepatic branches of the hepatic and portal veins [8]. Between the 10th and the 12th weeks, the left vitelline vein disappears, resulting in the formation of the terminal branch of the inferior vena cava, hepatic vein, and portal vein [8]. There is also potential for congenital IPSVSs to develop when the ductus venosus shunt fails to close, a cardiovascular defect known as patent ductus venosus (PDV) [9,10]. Closure of the ductus venosus begins immediately after birth and is completed within the first week of life. Failure of the ductus venosus to shut may result in hypoxemia, hepatic dysfunction, pulmonary hypertension, and hyperammonemic encephalopathy [11].

Until 2003, only approximately 50 cases of IPSVSs had been reported in the literature [12]. A study conducted in 2006 found that out of 25,579 asymptomatic adults, 6 (0.0235%) had an IPSVS upon imaging [13]. Another study found that the incidence of congenital IPSVSs was 1 in 30,000 births [14]; however, reported epidemiologic estimates for congenital intrahepatic portosystemic venous shunts should be interpreted with caution. Owing to the rarity of the condition, most prevalence data are derived from small screening studies, retrospective cohorts, or case-based literature rather than large population-based investigations. Furthermore, many patients remain asymptomatic, raising the possibility of underdiagnosis and suggesting that the true prevalence may be higher than currently reported [15].

**Figure 1:**
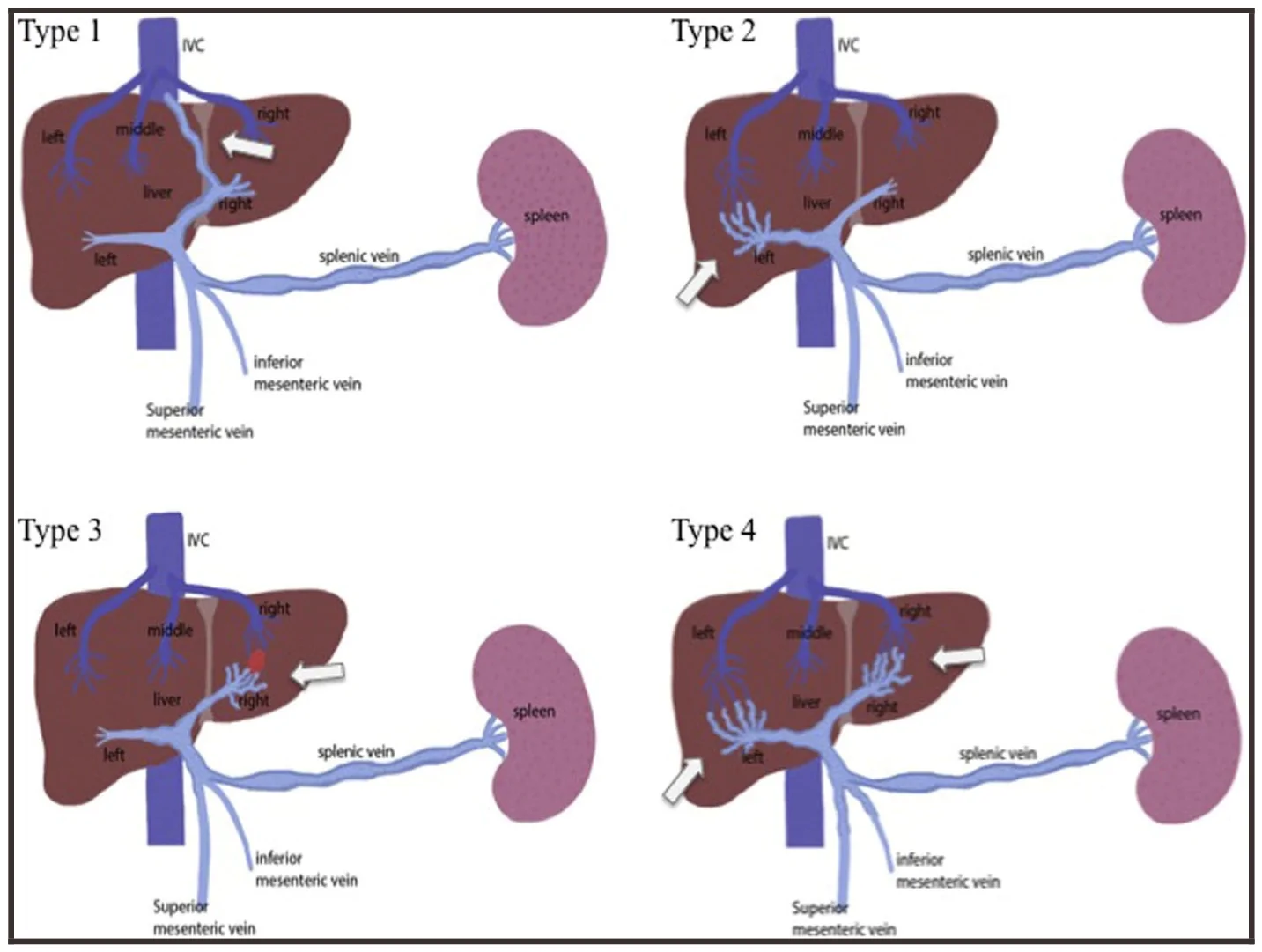
Image displaying the four types of IPSVSs according to Park’s classification. The malformation in each type of shunt is represented by the white arrows [17]. Image reproduced with permission from Reprinted with permission from Ref. [17]. Copyright 2018 Elsevier.

According to Park’s classification, IPSVSs can be categorized into four distinct types (Figure 1). Type 1 IPSVSs are the most common and occur when there is a vessel connecting the right portal vein and inferior vena cava. Type 2 IPSVS is characterized by single or multiple connections between the peripheral branches of the portal and hepatic veins in a single hepatic segment. Type 3 IPSVS occurs when an aneurysm connects the portal and hepatic veins, and Type 4 IPSVS is characterized by multiple connections between the portal and hepatic veins while involving both lobes of the liver [1,16]. Park’s classification is important because it categorizes congenital intrahepatic portosystemic venous shunts according to their vascular anatomy, providing a common language for diagnosis, reporting, and management. Understanding shunt morphology may also influence therapeutic decision-making and procedural planning.

In this article, we report two separate cases of symptomatic IPSVSs that varied in symptomatology from each other and were likely congenital, as they lacked the typical characteristics you would see in acquired IPSVSs that usually occur in the setting of trauma or cirrhosis [2,3,4]. The first case describes a patient who presented with complaints of chronic nausea and, upon imaging, was found to have an IPSVS. The second case involves a patient who presented with complaints of constipation, vomiting, unintentional weight loss, and hyperbilirubinemia, in which subsequent imaging was positive for the presence of an IPSVS. By presenting two patients with distinct clinical manifestations of congenital IPSVSs, this report illustrates the broad spectrum of disease presentation and reinforces the need for heightened clinical and radiologic awareness. These cases also highlight how accurate characterization of shunt anatomy can facilitate appropriate surveillance and treatment planning.

## 2. Case Presentation

*Case 1*

A female in her 30s, in no apparent distress, presented to Gastroenterology with the chief complaint of chronic nausea. The patient reported that her nausea and vomiting began seven years ago after her tubal ligation. She stated that about an hour after ingesting food, her stomach starts cramping and she vomits up undigested food. There was no nausea/vomiting after consuming liquids, but the patient reported 10/10 epigastric pain after eating. The patient denied fever or chills, marijuana use, odynophagia, chest pain, and hematuria. The patient’s current medications consisted of cyclobenzaprine, esomeprazole, promethazine, and diclofenac sodium. She had recently been prescribed promethazine, which she said helped with nausea.

**Figure 2:**
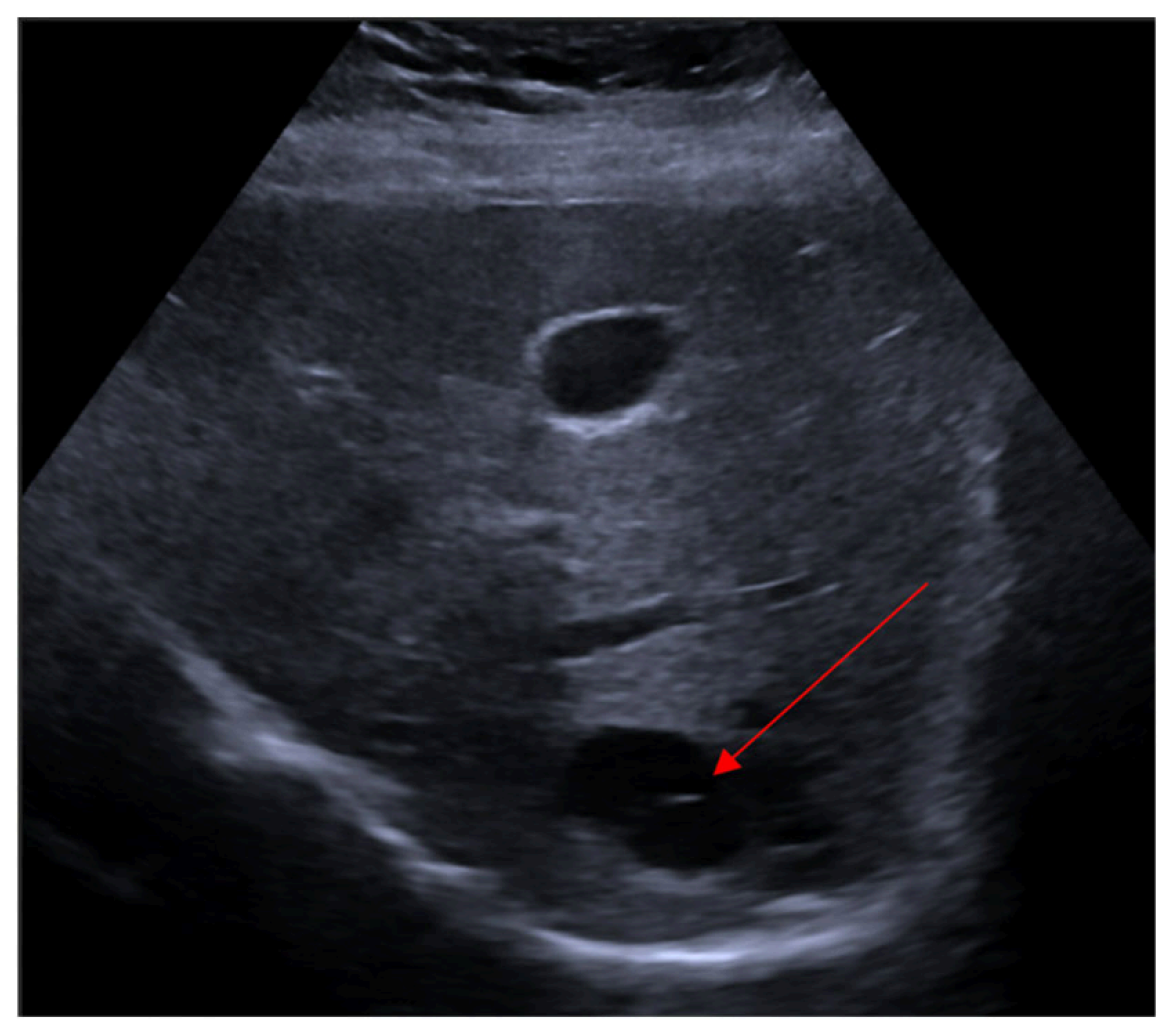
Abdominal ultrasound showing presence of a round cystic lesion in hepatic segment 6 with internal septation (red arrow).

**Figure 3:**
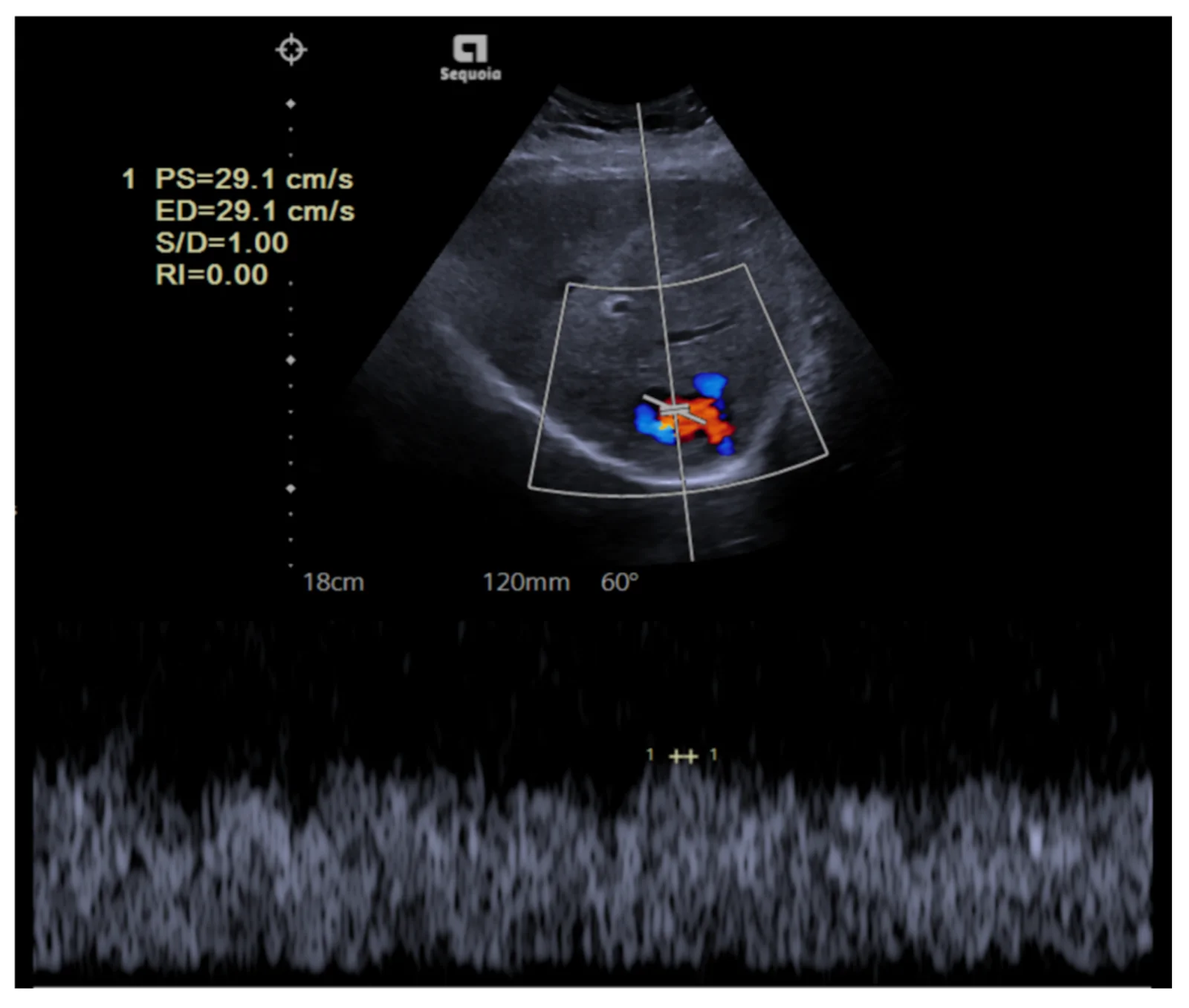
Color Doppler ultrasound of the cystic lesion showing an apparent connection with the hepatic venous system.

**Figure 4:**
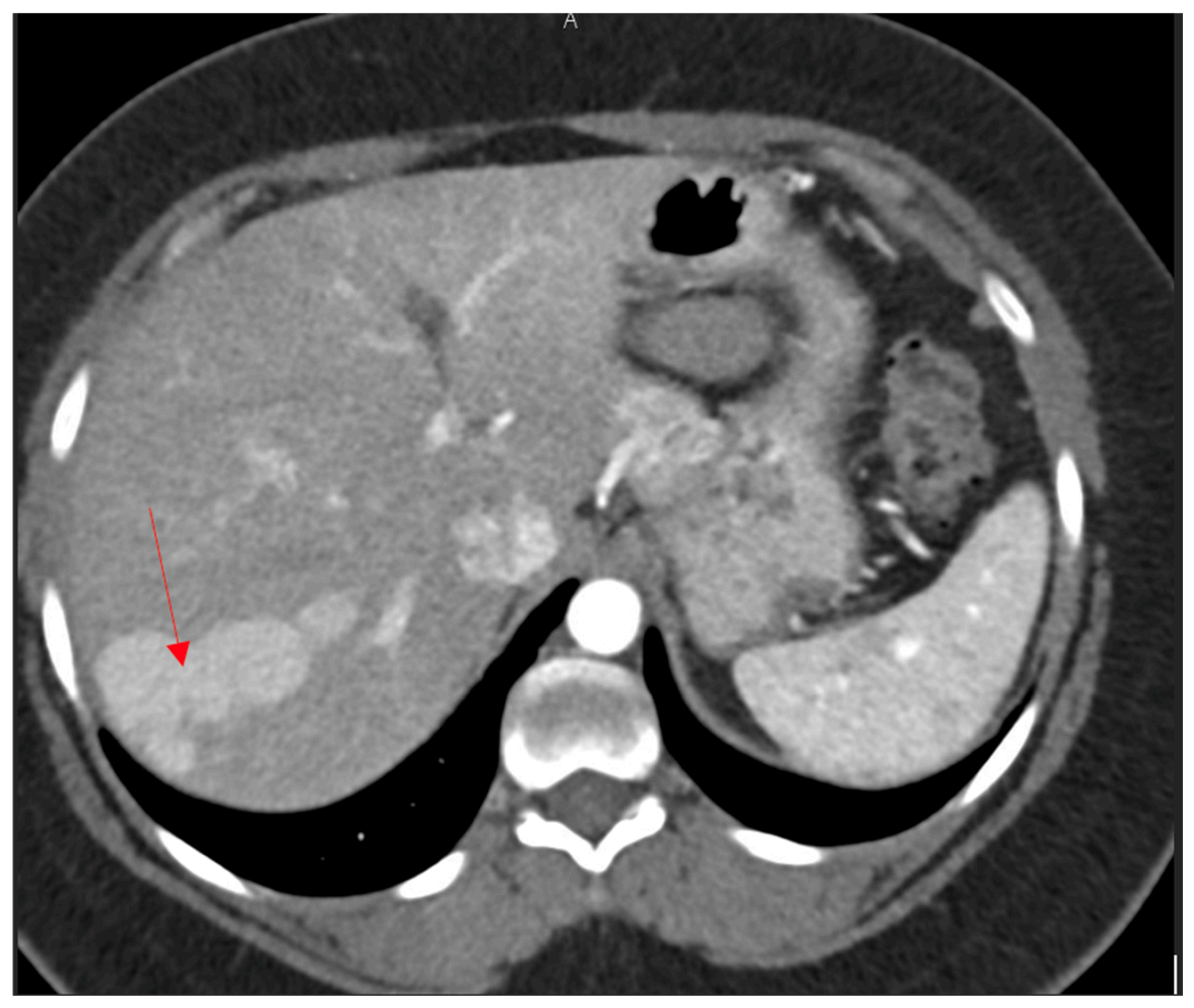
Axial CTA showing an intrahepatic portosystemic venous shunt (arrow) between the right portal and right hepatic veins.

The patient’s blood work was unremarkable. An ultrasound of the abdomen showed a round cystic lesion with internal septation measuring approximately 2.4 × 1.9 cm in hepatic segment 6 (Figure 2). On color Doppler, the lesion filled up entirely with color and appeared to connect with the hepatic venous system (Figure 3). The liver was normal in size, and there was no intrahepatic biliary dilatation or free fluid present within the abdomen. A computed tomography angiography (CTA) was positive for an IPSVS between the right portal and right hepatic veins (Figure 4). Embolization using an Amplatzer 2 plug to embolize the distal right hepatic vein just proximal to the portosystemic fistula was decided for treatment as this would redirect blood flow back through the normal portal venous system, restore hepatic perfusion, and reduce the potential physiologic consequences of portal blood bypassing the liver.

**Figure 5:**
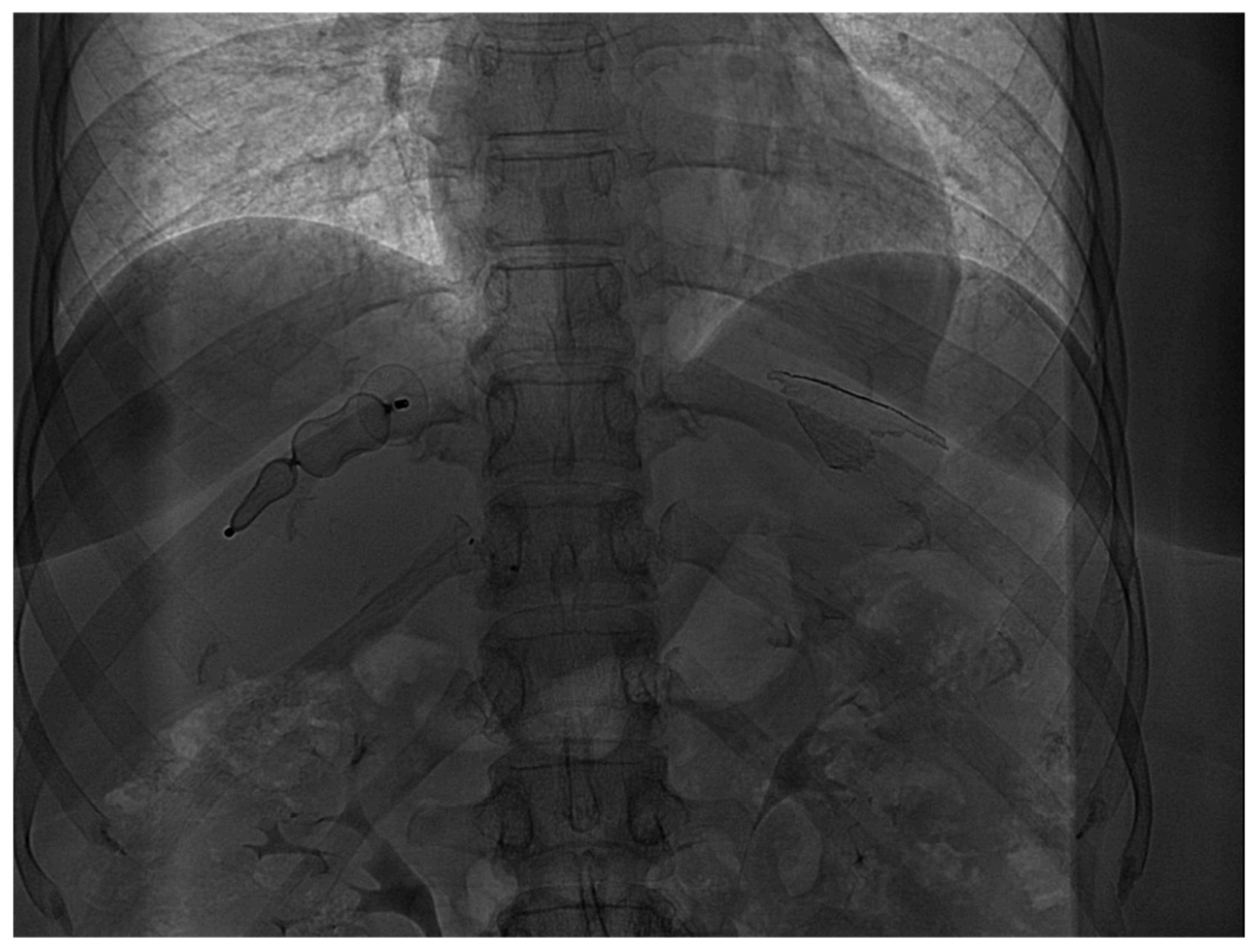
Both vascular plugs in stable position on post-embolization abdominal fluoroscopic scan.

**Figure 6:**
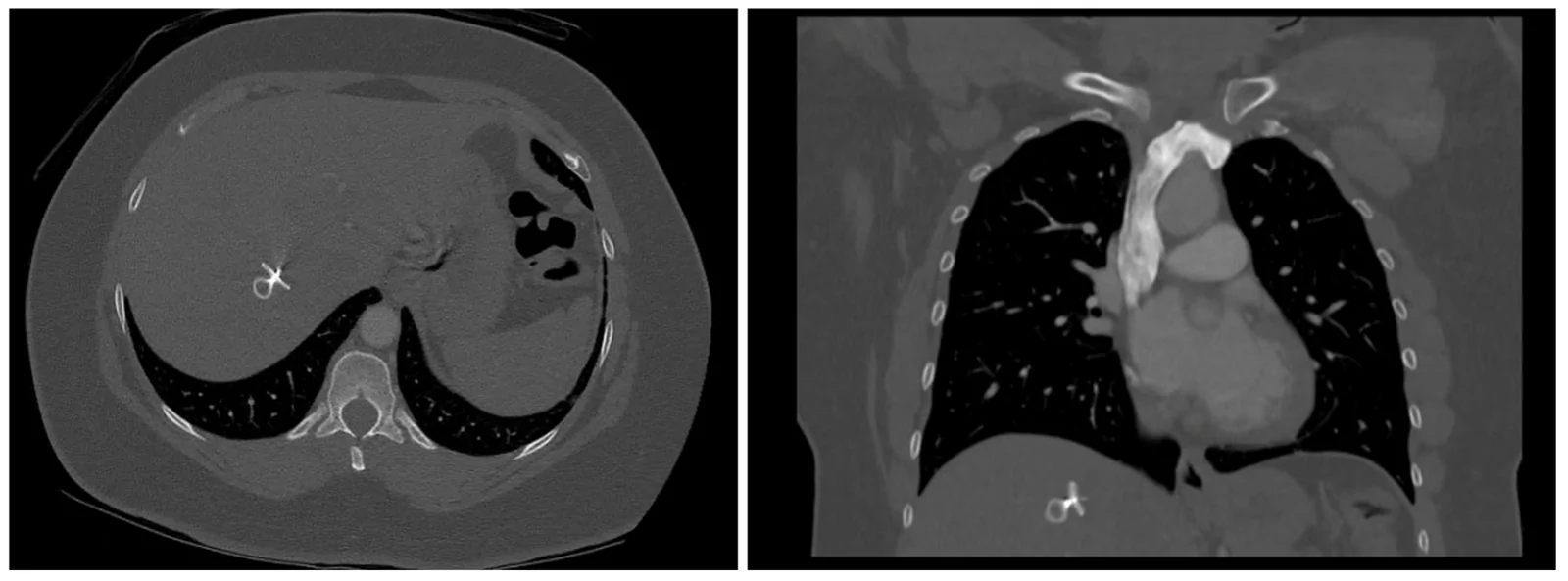
CT performed 20 days after surgery showing presence of vascular plugs in the liver.

Catheterization of the right hepatic vein was performed from a right internal jugular venous approach, and a digital subtraction angiography (DSA) was performed. With fluoroscopic guidance, measurement of the hepatic vein segment length and diameter for the plug placement location was obtained, and a larger Amplatzer vascular plug was inserted and placed at the hepatic outlet of the fistula. DSA evaluation of the fistula through the portal sheath showed a significant decrease in the fistula flow and increase in the right portal branch and left portal vein visualization. A smaller Amplatzer vascular plug was inserted into the left portal vein branch for hemostasis. To clarify, two plugs were used in this procedure, but each served different purposes. The larger Amplatzer vascular plug was deployed at the hepatic venous outlet of the fistula to occlude the portosystemic shunt, whereas the smaller plug was subsequently placed in the left portal venous access tract to achieve hemostasis following sheath removal. A post embolization fluoroscopic scan of the abdomen showed both vascular plugs in stable position (Figure 5). This was reiterated again 20 days after the procedure with a CT scan (Figure 6). The patient tolerated the procedure well with no complications and was discharged the next day after overnight observation. The patient was closely followed by the gastroenterology service and her primary care physician after embolization of the IPSVS. The patient reported improvement in her nausea, vomiting, and abdominal discomfort at her 1-month, 6-month, and 1-year follow-up appointments. Lab values remained grossly normal throughout that time.

*Case 2*

**Figure 7:**
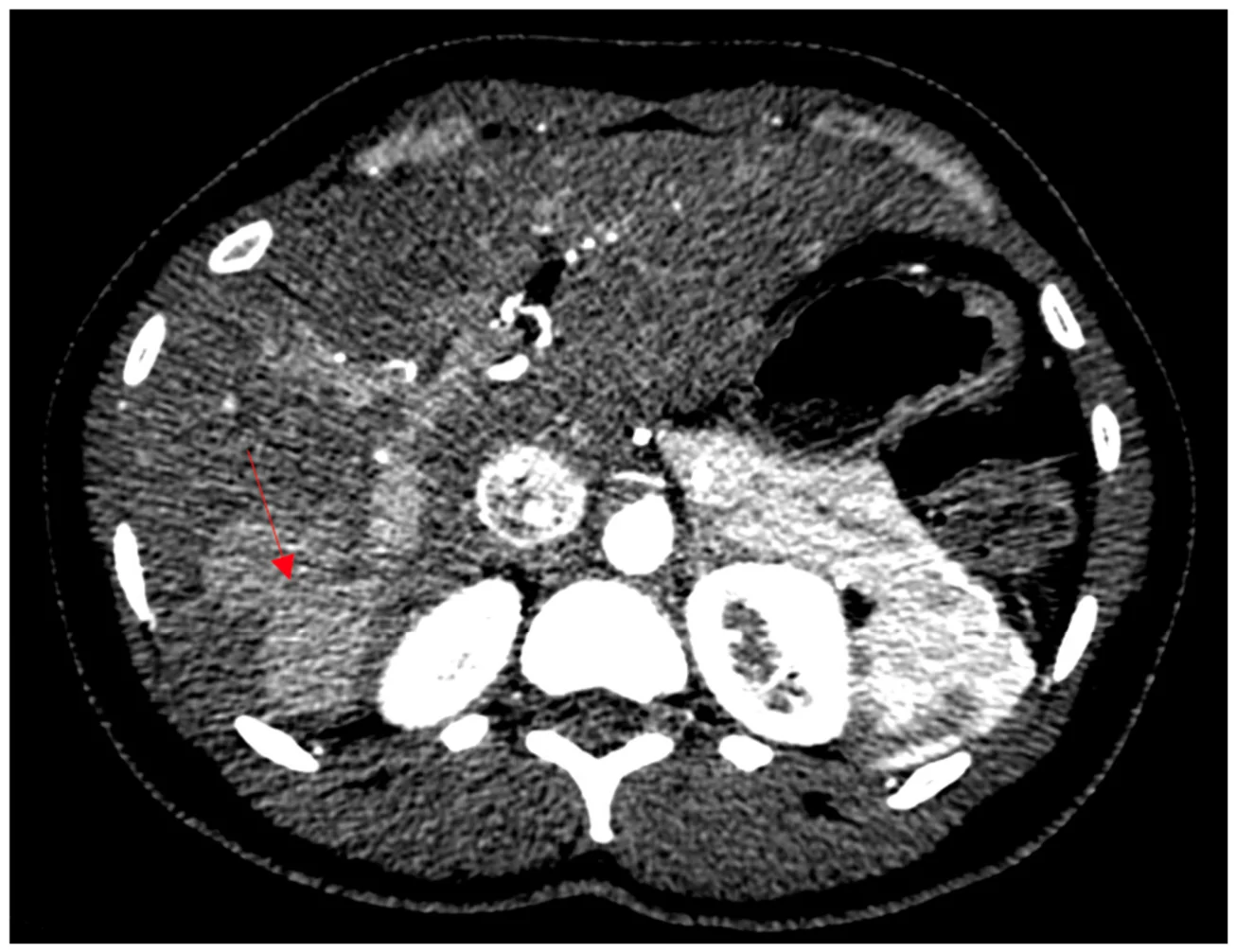
Axial CT showing presence of intrahepatic portosystemic venous shunt (arrow) between the posterior branch of the right portal vein and the right hepatic vein.

**Figure 8:**
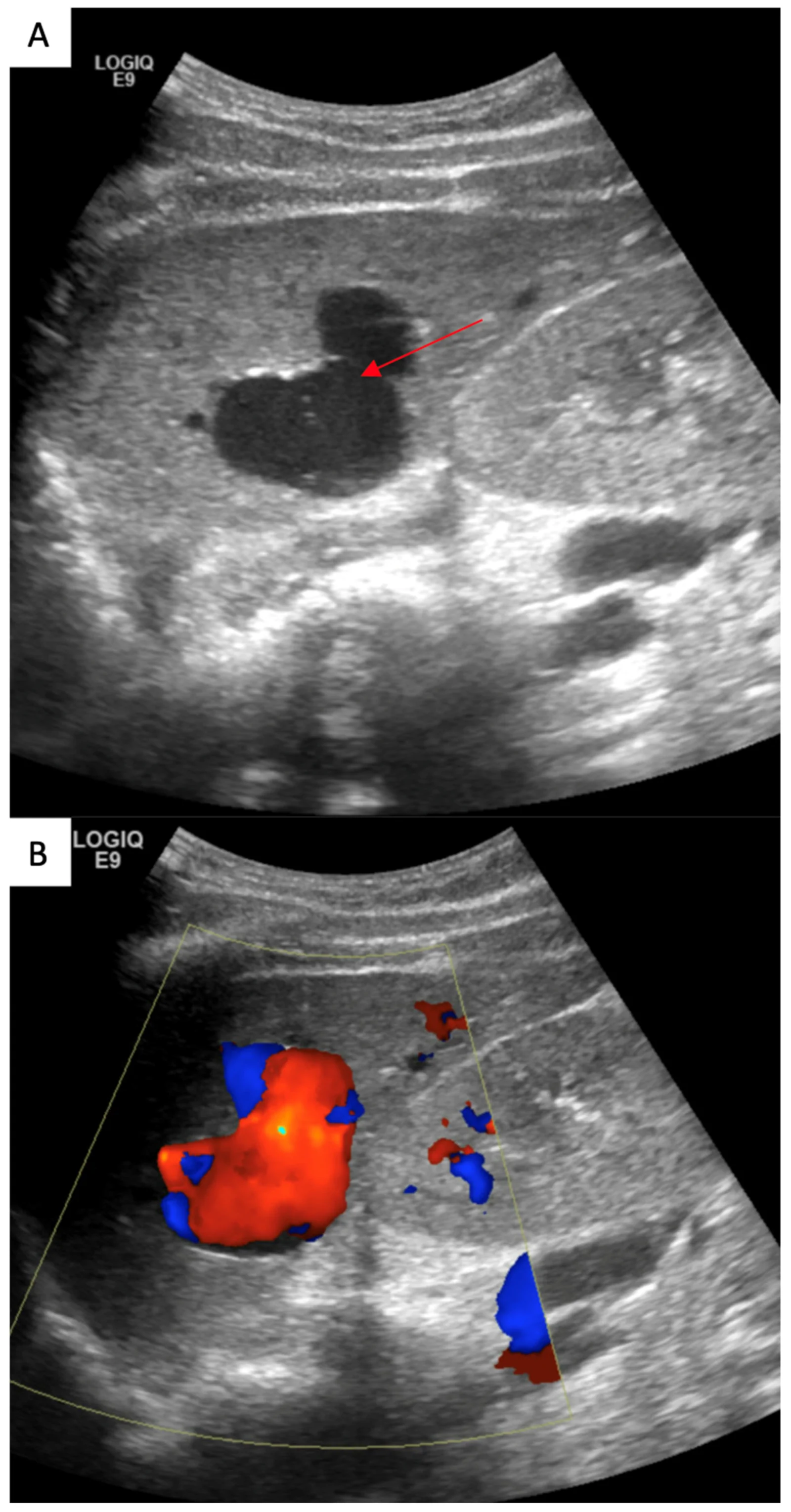
(**A**) Abdominal ultrasound, without Doppler, showing presence of an intrahepatic portosystemic venous shunt (arrow); (**B**) Abdominal ultrasound, with Doppler, showing presence of an intrahepatic portosystemic venous shunt.

**Figure 9:**
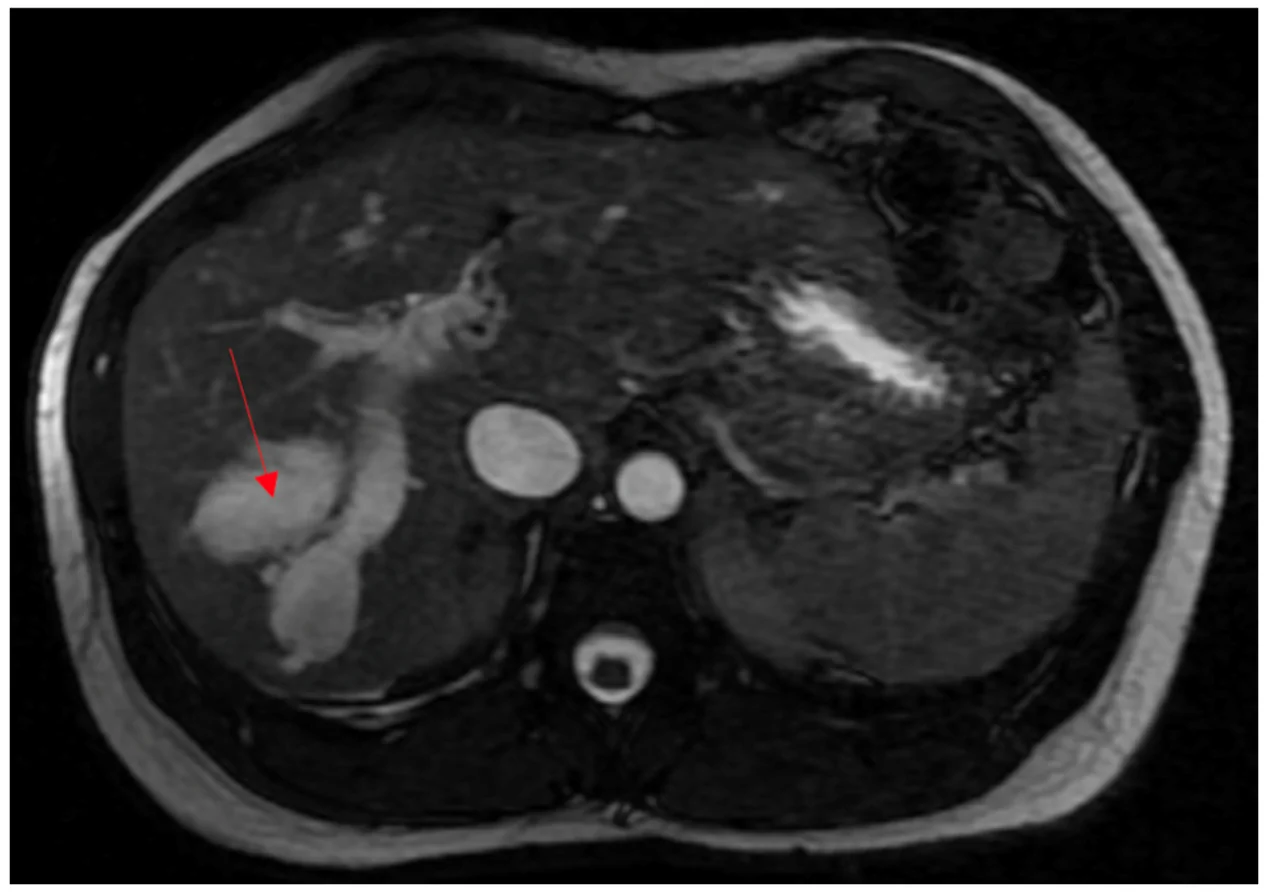
Axial view of MRI showing presence of an intrahepatic portosystemic venous shunt (arrow).

A female in her 30s presented to the hospital with complaints of intermittent constipation along with abdominal pain and vomiting but no nausea. The patient denied the use of nonsteroidal anti-inflammatory drugs but said that she had been taking polyethylene glycol 3350 for two weeks, which helped her constipation. Unintentional weight loss of 30 lbs. over the last six months was also reported by the patient, and mild hyperbilirubinemia, 1.4 mg/dL, was noted four months earlier. A CT of the abdomen and pelvis with contrast showed the presence of a large IPSVS between the posterior branch of the right portal vein and the right hepatic vein (Figure 7). This was followed by an ultrasound and magnetic resonance imaging (MRI) of the abdomen, both redemonstrating a likely congenital IPSVS (Figure 8 and Figure 9).

**Figure 10:**
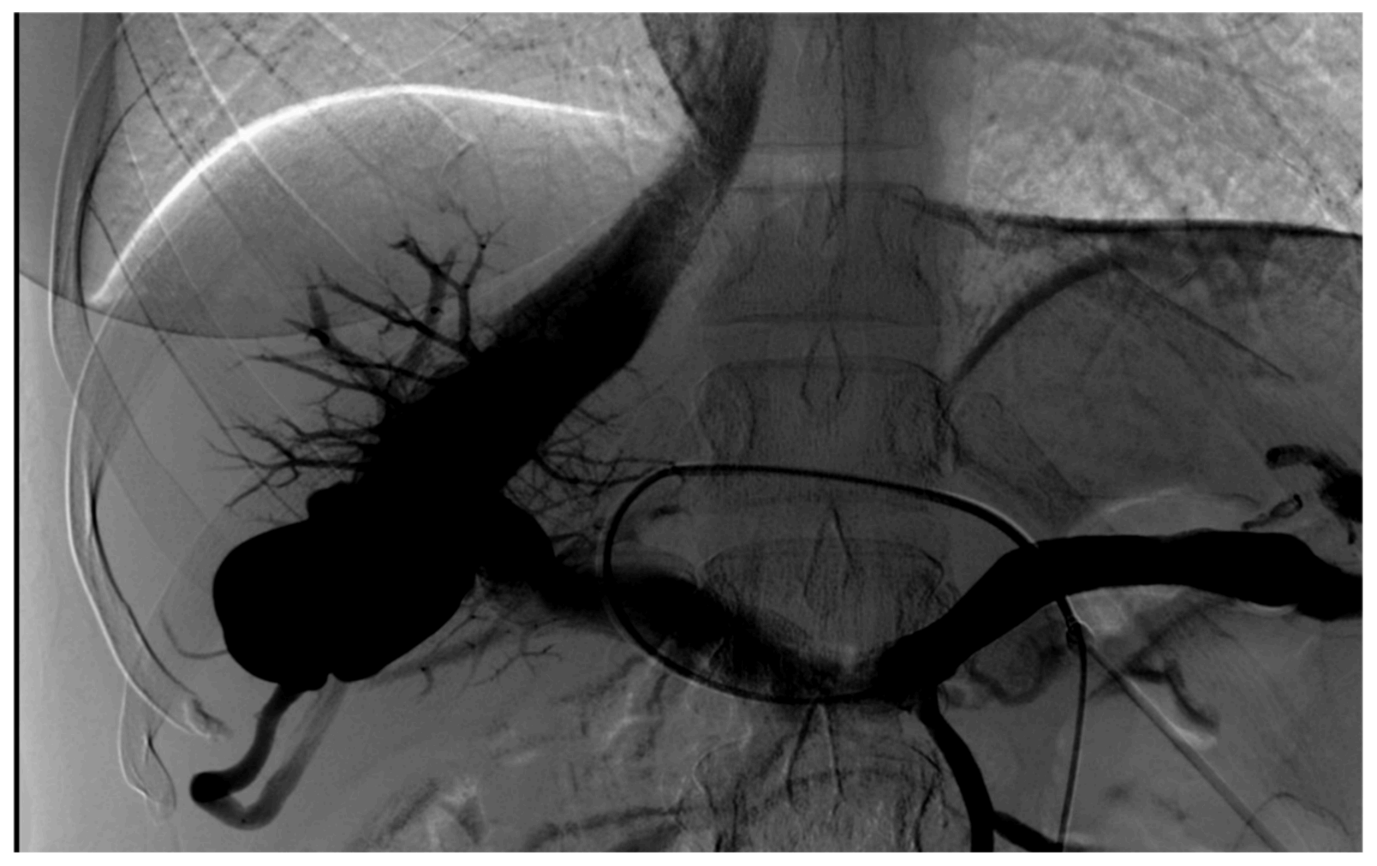
Portogram showing successful embolization of the shunt.

**Figure 11:**
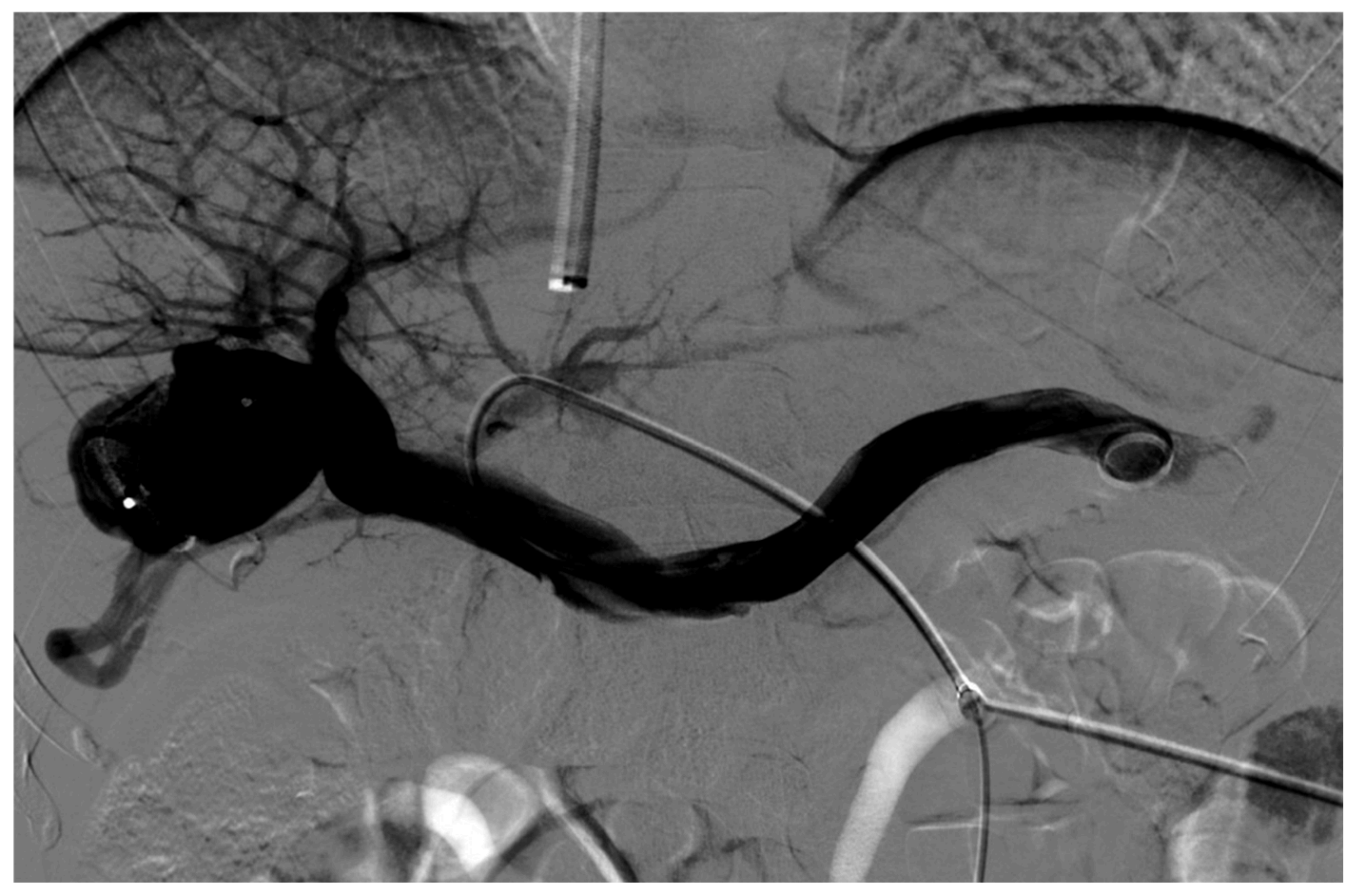
Both vascular plugs in stable position on post-embolization abdominal fluoroscopic scan.

**Figure 12:**
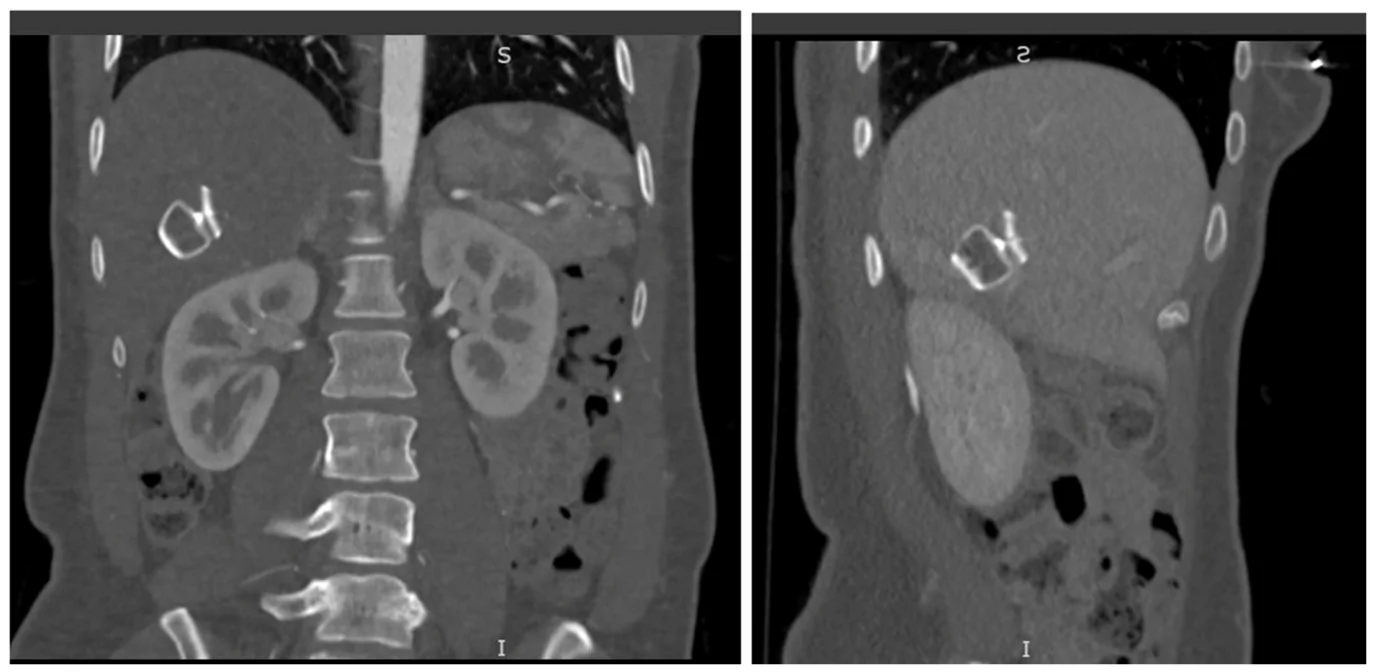
CT imaging three months after the procedure showing vascular plugs still in place with successful occlusion of the portal venous fistula.

Catheterization of the right hepatic vein was performed as described in the previous case, and a right hepatic venogram was performed to confirm location. The distal right hepatic vein just proximal to the portosystemic fistula was embolized using an Amplatzer plug. Successful embolization was confirmed with a portogram through the percutaneous portal vein access (Figure 10). A smaller Amplatzer plug was then placed as described before for the same reason. An abdominal fluoroscopic scan performed immediately after the procedure and CT imaging performed three months later showed the vascular plugs to be in stable position (Figure 11 and Figure 12). The patient was followed by the interventional radiology service at 6 months and 12 months post-operatively, with resolution of symptoms reported and no further derangements in her labs.

## 3. Discussion

IPSVSs are rare abnormalities that occur when there is unusual communication between the portal and hepatic veins [1]. In this manuscript, we reported on two patients who were diagnosed with an IPSVS and successfully treated with embolization. This report serves as a reminder of the various clinical presentations that can be seen with this rare condition and how it is diagnosed. It also adds to the existing body of literature that embolization is a successful mode of treatment for IPSVSs. Additionally, the present cases are unique in their symptomatic presentation, as neither patient presented with the typical signs and symptoms you would usually see with IPSVSs, such as abnormal liver function tests, hepatic encephalopathy, and other findings related to portal hypertension.

Given the rarity of congenital IPSVSs, the available literature consists primarily of case reports, small case series, and a limited number of comprehensive reviews. Two notable contributions to the literature include the comprehensive review by Papamichail et al. [8] and the prenatal case series reported by Zhu et al. [18], which together provide important insights into the presentation, natural history, and outcomes of congenital IPSVSs. In their comprehensive review, Papamichail et al. summarized the embryology, classification, clinical manifestations, and management of both intrahepatic and extrahepatic shunts. The authors emphasized that IPSVSs are rare developmental vascular anomalies resulting from abnormal remodeling of the fetal vitelline and cardinal venous systems, with intrahepatic shunts representing a distinct subgroup that may undergo spontaneous closure during infancy. The review highlighted the broad clinical spectrum of disease, ranging from asymptomatic incidental findings to complications such as hyperammonemia, hepatic encephalopathy, hepatopulmonary syndrome, pulmonary hypertension, and benign or malignant hepatic tumors. Associated congenital anomalies, particularly cardiovascular abnormalities, were also frequently reported. The authors proposed that management should be guided by shunt anatomy, symptom burden, and the risk of long-term complications, with closure recommended for persistent shunts associated with significant clinical sequelae [8]. In a prenatal series of 19 fetuses with congenital IPSVSs, Zhu et al. found that most lesions originated from the left portal venous system and were diagnosed during the late second or third trimester. Fetal growth restriction was the most common associated prenatal finding (47.4%), followed by cardiac enlargement (21.1%). Postnatally, affected infants demonstrated a range of manifestations including transient hyperammonemia, hypoglycemia, cholestatic biochemical abnormalities, pulmonary hypertension, and hepatic nodules. Importantly, spontaneous closure occurred in most conservatively managed cases, with a mean closure time of approximately 8 months, supporting the generally favorable prognosis of isolated prenatal intrahepatic shunts while underscoring the need for postnatal surveillance for metabolic and vascular complications [18].

Symptoms related to IPSVSs are non-specific, and patients can present with a wide array of symptoms. Some people have no symptoms, and the shunt is discovered incidentally, while others develop significant problems depending on the size of the shunt and how much portal blood bypasses the liver [8,19]. Hepatic encephalopathy is one of the most prominent clinical manifestations, and adults often present with neurologic symptoms resembling chronic liver disease [7,8,20]. Gastrointestinal symptoms, such as weight loss, abdominal pain, nausea, and vomiting, are less specific [8], thus adding another interesting and novel detail to our two cases. Mesenteric venous congestion refers to increased pressure or impaired drainage in the veins that carry blood away from the intestines and into the portal venous system and is likely a contributor to the gastrointestinal symptoms in the setting of IPSVSs. In normal settings, blood flows from the intestines through the mesenteric veins into the portal vein. The liver receives and processes this blood before it returns to the systemic circulation. When venous drainage is impaired or portal pressures are abnormal, such as in IPSVSs, blood can pool into intestinal veins and cause the bowel wall to become edematous, thus leading to less efficient digestion and absorption as seen in the present cases [21,22]; additionally, stretching of the bowel wall and altered motility can trigger gastrointestinal symptoms [21,22]. The symptomatic burden experienced by our patients is what led us to choose embolization over conservative management.

Since there is an absence of specific clinical symptomologies that present with congenital IPSVSs, imaging is paramount when diagnosing this condition. Most imaging techniques successfully show the existence of a fistula between the hepatic and portal vein if an IPSVS is present [23,24]. However, this abnormal communication is not always seen on imaging, in which secondary findings, such as abnormal blood pooling from a dilated portal branch, often point towards the presence of an IPSVS [17,24]. Signs of this condition are usually first seen with Doppler ultrasound, a non-invasive technique that measures blood flow through body vessels [23]. CT and MRI are valuable in confirming the diagnosis, with MRI usually being performed first due to no radiation exposure to the patient as compared to CT [23]. In our report, imaging was key in diagnosing both patients. In the first case, an ultrasound of the abdomen first showed evidence of an IPSVS being present. A CTA was then used to confirm the diagnosis. In the second case, a diagnosis of an IPSVS was made after it was seen on a CT of the abdomen and pelvis with contrast. Ultrasound and MRI were then used for further evaluation, with both showing the existence of an IPSVS. The use of multiple imaging modalities was performed to ensure accurate visualization and characterization of these shunts so that pre-operative planning could be performed. The diagnostic advantages of each of these imaging types are summarized in Table 1.

Treatment can differ between asymptomatic and symptomatic IPSVSs. For patients presenting with asymptomatic IPSVSs, which are the majority of IPSVSs and are usually discovered incidentally upon imaging, there is no definitive treatment plan [1,8]. Although endovascular techniques have proven beneficial in treating symptomatic patients with IPSVSs, it is not agreed upon if the risks of performing a procedure on an asymptomatic patient outweigh the risks of not having the procedure, especially when there is still not a clear understanding as to why many IPSVSs remain asymptomatic [1,25]. For symptomatic IPSVSs, it is well concluded that transcatheter embolization, which is performed to interrupt the abnormal communication and restore blood flow to the liver, is highly effective and recommended [1,8,12,26]. The use of coils has been recommended for smaller shunts, while a vascular plug, such as an Amplatzer, has been advised for larger shunts [8]. If left untreated, potential long-term sequelae include hepatic encephalopathy, pulmonary hypertension, and/or heart failure [25,27,28]. For both symptomatic and asymptomatic congenital IPSVSs diagnosed prenatally or early during infancy, immediate treatment is usually bypassed since spontaneous regression of the shunt commonly occurs within 1 year after birth [8]. In the present cases, both individuals presented with symptomatic IPSVS that were likely congenital in nature and successfully treated with transcatheter embolization using a vascular plug, meaning our treatment of choice did not differ from previously reported cases, as vascular plugs are commonly used for treatment of these shunt types [6,29].

## 4. Conclusions

In this manuscript, we presented two cases of symptomatic IPSVSs that were likely congenital in etiology. The shunts were diagnosed using a combination of ultrasound, CT, and MRI, followed by successful treatments with transcatheter embolization procedures. This report highlights the variance in symptomatology that can be seen in patients with symptomatic IPSVSs, as each case presented with different symptomatic features, while also reinforcing the notion that the use of different medical imaging modalities is of high importance when trying to make a diagnosis of IPSVSs. In addition, this article also adds to the existing literature that transcatheter embolization is a valuable therapeutic approach for symptomatic IPSVSs.

## Figures and Tables

**Table 1:** Diagnostic advantages and limitations of Doppler ultrasound, CT angiography, and MRI in the evaluation of congenital intrahepatic portosystemic venous shunts.

Feature	Doppler Ultrasound	CT Angiography (CTA)	MRI/MR Angiography (MRA)
**Initial detection**	Excellent first-line test	Good	Good
**Shows abnormal vascular communication**	Usually yes	Excellent	Excellent
**Shows direction of blood flow**	Best modality	Limited	Moderate (with phase-contrast techniques)
**Measures flow velocity**	Best modality	No	Limited
**Defines shunt anatomy**	Moderate	Excellent	Excellent
**Identifies feeding/draining vessels**	Moderate	Excellent	Excellent
**Detects small shunts**	Operator-dependent	Excellent	Very good
**3D vascular mapping**	Limited	Excellent	Excellent
**Pre-embolization planning**	Helpful	Often preferred	Excellent alternative
**Radiation exposure**	None	Yes	None

## Data Availability

The original data presented in the study are included in the article, further inquiries can be directed to the corresponding author.

## Abbreviations

The following abbreviations are used in this manuscript:

MRI	magnetic resonance imaging
IPSVSs	intrahepatic portosystemic venous shunts
CTA	computed tomography angiography
DSA	digital subtraction angiography
